# Predicting Abraham model solvent coefficients

**DOI:** 10.1186/s13065-015-0085-4

**Published:** 2015-03-22

**Authors:** Jean-Claude Bradley, Michael H Abraham, William E Acree, Andrew SID Lang

**Affiliations:** Department of Chemistry, Drexel University, Philadelphia, PA 19104 USA; Department of Chemistry, University College London, Gordon Street, WC1H 0AJ London, UK; Department of Chemistry, University of North Texas, 1155 Union Cir, Denton, TX 76203 USA; Department of Computing and Mathematics, Oral Roberts University, 7777 S. Lewis Avenue, Tulsa, OK 74171 USA

**Keywords:** Abraham general solvation model, Solvent coefficients, Sustainable solvents, Solvent replacement, Partition coefficients, Solubility

## Abstract

**Background:**

The Abraham general solvation model can be used in a broad set of scenarios involving partitioning and solubility, yet is limited to a set of solvents with measured Abraham coefficients. Here we extend the range of applicability of Abraham’s model by creating open models that can be used to predict the solvent coefficients for all organic solvents.

**Results:**

We created open random forest models for the solvent coefficients e, s, a, b, and v that had out-of-bag R^2^ values of 0.31, 0.77, 0.92, 0.47, and 0.63 respectively. The models were used to suggest sustainable solvent replacements for commonly used solvents. For example, our models predict that propylene glycol may be used as a general sustainable solvent replacement for methanol.

**Conclusion:**

The solvent coefficient models extend the range of applicability of the Abraham general solvation equations to all organic solvents. The models were developed under Open Notebook Science conditions which makes them open, reproducible, and as useful as possible.

**Graphical Abstract:**
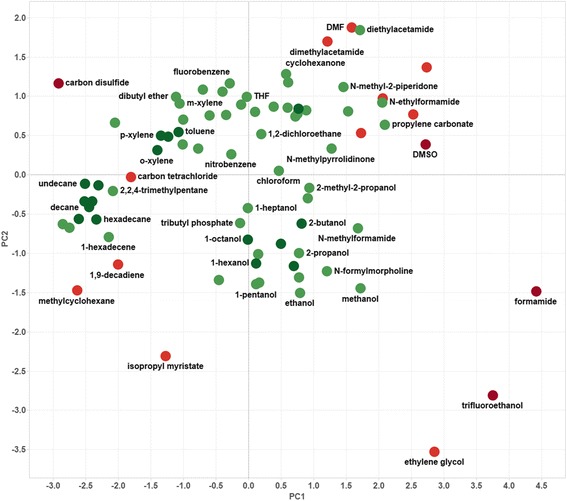
Chemical space for solvents with known Abraham coefficients.

**Electronic supplementary material:**

The online version of this article (doi:10.1186/s13065-015-0085-4) contains supplementary material, which is available to authorized users.

## Background

The Abraham model was developed and is widely used to predict partition coefficients for both conventional organic solvents [1-11] and ionic liquid solvents [12,13], for the partitioning of drug molecules between blood and select body organs [14-18], and for partitioning into micelles [19] and for prediction of enthalpies of solvation in organic solvents [20] and ionic organic liquids [21]. The Abraham model is based on the linear free energy relationship (LFER)

1 $$ log\ P = c+e\ E+s\ S+a\ A+b\ B+v\ V $$

where logP is the solvent/water partition coefficient. Under reasonable conditions, this model can also be used to predict the solubility of organic compounds in organic solvents [22] as follows

2 $$ log\ {S}_s= log\ {S}_w+c+e\ E+s\ S+a\ A+b\ B+v\ V $$

where *S*_*s*_ is the molar concentration of the solute in the organic solvent, *S*_*w*_ is the molar concentration of the solute in water, (c, e, s, a, b) are the solvent coefficients, and (E, S, A, B, V) are the solute descriptors: E is the solute excess molar refractivity in units of (cm^3/mol)/10, S is the solute dipolarity/polarizability, A and B are the overall or summation hydrogen bond acidity and basicity, and V is the McGowan characteristic volume in units of (cm^3/mol)/100.

The solvent coefficients are obtained by linear regression using experimentally determined partitions and solubilities of solutes with known Abraham descriptors. Traditionally, the intercept c is allowed to float and is assumed to encode information not characterized by the other solvent-solute interaction terms. However, for some partitioning systems the value of *c* can vary greatly depending upon the training-set used [23]. This makes it difficult to directly compare different solvents by examining their solvent coefficients. Van Noort has even suggested that the *c*-coefficient be derived directly from structure before the other coefficients are determined [24]. A problem with this suggestion is that the c-coefficient depends on the standard state. Partition coefficients can be expressed in concentration units of molarity and mole fractions, and the numerical value of the *c*-coefficient will be different for each concentration unit. Abraham model correlations considered in this study have partition coefficients expressed in concentration units of molarity.

To date, solvent coefficients have been determined for over 90 commonly used solvents (Additional file 1), and group contribution methods have been developed to approximate all coefficients for certain classes of solvents that do not have published solvent coefficients [25,26]. The solvent coefficients in the supporting material pertain to dry solvents, or solvents that take up very little water (hexane, toluene, etc.). This study expands the applicability of the Abraham model by developing open models, using open descriptors from the Chemistry Development Kit (CDK) [27] that can be used to predict the Abraham solvent coefficients of any organic solvent directly from structure.

## Procedure

In order to directly compare various solvents, it is advantageous to first recalculate the solvent coefficients with the c-coefficient equal zero. This was accomplished by using equation (1) to calculate the log *P* values for 2144 compounds from our Open Data database of compounds with known Abraham descriptors [28] and then by regressing the results against the following equation

3 $$ log\ P = {e}_0\ E+{s}_0\ S+{a}_0\ A+{b}_0\ B+{v}_0\ V $$

where the subscript-zero indicates that *c* = 0 has been used in the regression [29]. As an informational note one could have set the *c*-coefficient of a given solvent equal to a calculated average value determined from numerical *c*-coefficients of solvents similar to the solvent under consideration. For example, the *c*-coefficient of all alkane solvents could be set equal to *c* = 0.225, which is the average value for the *c*-coefficients of the 13 alkane and cycloalkane solvents for which log *P* correlations have been determined. While average values could be used for several solvents, there is the problem of what value to use in the case of solvents for which a similar solvent log *P* solvent is not available. Abraham model correlations are available for two dialkyl ethers (e.g., diethyl ether and dibutyl ether) and for several alcohols, but not for alkoxyalcohols (e.g., 2-ethoxyethanol, 2-propoxyethanol, 2-butyoxyethanol) which contain both an ether and hydroxyl alcohol group. Our intended solvent set in the present communication includes the alternative “green” solvents, and there a number of solvents in this group that contain multi-functional groups. For several of the solvents on the list of alternative “green” solvents, such as 1,3-dioxan-5-ol, 1,3-dioxolane-4-methanol, 3-hydroxypropionic acid, 5-(hydroxymethyl)furfural, ethyl lactate, furfuryl alcohol, and other solvents, there are no similar solvents having a Abraham model log *P* correlation. To treat all solvents equally we have elected to set *c* = 0 in this study.

Table 1 lists the original solvent coefficients together with the *c* = 0 adjusted coefficients. Comparing the coefficients, we see, not surprisingly, the largest changes in coefficient values occur for solvents with *c*-values furthest away from zero (Additional file 1). What is intriguing is that all the coefficients move consistently the same way. That is, solvents with negative c-values all saw an increase in *e* and *b* (and a decrease in *s*, *a*, and *v*) when recalculated, whereas solvents with positive c-values all saw an increase in *s*, a, and v (and decrease in e and b).

**Table 1:** **Solvent coefficients**

**c**	**e**	**s**	**a**	**b**	**v**	**Solvent**	**e**_**0**_	**s**_**0**_	**a**_**0**_	**b**_**0**_	**v**_**0**_
0.351	0.223	−0.150	−1.035	−4.527	3.972	methyl acetate	0.195	−0.068	−0.924	−4.571	4.152
0.328	0.369	−0.446	−0.700	−4.904	4.150	ethyl acetate	0.343	−0.369	−0.597	−4.945	4.319
0.248	0.356	−0.501	−0.867	−4.973	4.281	butyl acetate	0.336	−0.443	−0.788	−5.005	4.409
−0.605	0.930	−1.153	−1.682	−4.093	4.249	isopropyl myristate	0.977	−1.295	−1.870	−4.018	3.939
0.090	0.205	−0.172	1.305	−4.589	3.833	N-methylacetamide	0.197	−0.151	1.335	−4.601	3.880
0.284	0.128	−0.442	1.180	−4.728	3.856	N-ethylacetamide	0.105	−0.375	1.269	−4.764	4.002
−0.271	0.084	0.209	0.915	−5.003	4.557	dimethylacetamide	0.105	0.145	0.832	−4.970	4.419
0.213	0.034	0.089	1.342	−5.084	4.088	N,N-diethylacetamide	0.017	0.139	1.409	−5.111	4.198
−0.171	0.070	0.308	0.589	−3.152	2.432	formamide	0.083	0.268	0.537	−3.132	2.345
0.114	0.407	−0.287	0.542	−4.085	3.471	N-methylformamide	0.398	−0.260	0.579	−4.100	3.530
0.220	0.034	−0.166	0.935	−4.589	3.730	N-ethylformamide	0.016	−0.114	1.005	−4.617	3.843
−0.305	−0.058	0.343	0.358	−4.865	4.486	DMF	−0.034	0.271	0.264	−4.828	4.330
0.332	0.302	−0.436	0.358	−4.902	3.952	dibutylformamide	0.275	−0.358	0.462	−4.944	4.123
0.276	0.334	−0.714	0.243	−3.320	3.549	methanol	0.312	−0.649	0.330	−3.355	3.691
0.222	0.471	−1.035	0.326	−3.596	3.857	ethanol	0.453	−0.983	0.396	−3.623	3.971
0.243	0.213	−0.575	0.262	−3.450	3.545	ethanol/water(90:10)vol	0.193	−0.518	0.339	−3.481	3.670
0.172	0.175	−0.465	0.260	−3.212	3.323	ethanol/water(80:20)vol	0.161	−0.424	0.314	−3.233	3.411
0.063	0.085	−0.368	0.311	−2.936	3.102	ethanol/water(70:30)vol	0.079	−0.353	0.331	−2.944	3.134
−0.040	0.138	−0.335	0.293	−2.675	2.812	ethanol/water(60:40)vol	0.141	−0.344	0.281	−2.670	2.792
−0.142	0.124	−0.252	0.251	−2.275	2.415	ethanol/water(50:50)vol	0.135	−0.285	0.207	−2.257	2.342
−0.221	0.131	−0.159	0.171	−1.809	1.918	ethanol/water(40:60)vol	0.148	−0.211	0.103	−1.782	1.805
−0.269	0.107	−0.098	0.133	−1.316	1.414	ethanol/water(30:70)vol	0.128	−0.161	0.049	−1.283	1.276
−0.252	0.043	−0.040	0.096	−0.832	0.916	ethanol/water(20:80)vol	0.063	−0.099	0.017	−0.801	0.787
−0.173	−0.023	−0.001	0.065	−0.372	0.454	ethanol/water(10:90)vol	−0.009	−0.042	0.011	−0.350	0.365
0.000	0.000	0.000	0.000	0.000	0.000	water	0.000	0.000	0.000	0.000	0.000
0.139	0.405	−1.029	0.247	−3.767	3.986	1-propanol	0.393	−0.996	0.291	−3.785	4.058
0.099	0.343	−1.049	0.406	−3.827	4.033	2-propanol	0.335	−1.026	0.438	−3.839	4.084
0.188	0.354	−1.127	0.016	−3.568	3.968	2-methyl-1-propanol	0.339	−1.083	0.076	−3.592	4.065
0.211	0.171	−0.947	0.331	−4.085	4.109	2-methyl-2-propanol	0.154	−0.897	0.398	−4.112	4.218
0.165	0.401	−1.011	0.056	−3.958	4.044	1-butanol	0.388	−0.972	0.108	−3.979	4.129
0.127	0.253	−0.976	0.158	−3.882	4.114	2-butanol	0.242	−0.946	0.199	−3.898	4.179
0.073	0.360	−1.273	0.090	−3.770	4.399	3-methyl-1-butanol	0.354	−1.256	0.113	−3.779	4.437
0.150	0.536	−1.229	0.141	−3.864	4.077	1-pentanol	0.524	−1.194	0.188	−3.883	4.154
0.115	0.455	−1.331	0.206	−3.745	4.201	2-pentanol	0.445	−1.304	0.243	−3.759	4.260
0.115	0.492	−1.164	0.054	−3.978	4.131	1-hexanol	0.483	−1.137	0.091	−3.993	4.191
0.035	0.398	−1.063	0.002	−4.342	4.317	1-heptanol	0.395	−1.055	0.014	−4.347	4.335
−0.034	0.489	−1.044	−0.024	−4.235	4.218	1-octanol	0.491	−1.052	−0.034	−4.231	4.201
−0.058	0.616	−1.319	0.026	−4.153	4.279	1-decanol	0.620	−1.333	0.009	−4.146	4.250
−0.096	0.148	−0.841	−0.438	−4.040	4.125	octadecanol	0.155	−0.864	−0.467	−4.028	4.076
0.369	0.386	−1.568	−3.535	−5.215	4.514	pentane	0.357	−1.481	−3.419	−5.261	4.704
0.333	0.560	−1.710	−3.578	−4.939	4.463	hexane	0.533	−1.632	−3.473	−4.981	4.634
0.297	0.643	−1.755	−3.571	−4.946	4.488	heptane	0.619	−1.685	−3.477	−4.983	4.641
0.231	0.738	−1.840	−3.585	−4.907	4.502	octane	0.719	−1.786	−3.512	−4.936	4.621
0.240	0.619	−1.713	−3.532	−4.921	4.482	nonane	0.600	−1.657	−3.457	−4.951	4.606
0.186	0.722	−1.741	−3.449	−4.970	4.476	decane	0.707	−1.697	−3.390	−4.993	4.572
0.058	0.603	−1.661	−3.421	−5.120	4.619	undecane	0.598	−1.647	−3.402	−5.128	4.649
0.114	0.668	−1.644	−3.545	−5.006	4.459	dodecane	0.659	−1.617	−3.509	−5.021	4.518
0.087	0.667	−1.617	−3.587	−4.869	4.433	hexadecane	0.660	−1.596	−3.560	−4.880	4.478
0.320	0.511	−1.685	−3.687	−4.811	4.399	2,2,4-trimethylpentane	0.485	−1.610	−3.586	−4.851	4.564
0.104	0.615	−1.796	−3.070	−4.291	4.518	1,9-decadiene	0.606	−1.771	−3.037	−4.304	4.572
0.116	0.706	−1.616	−3.181	−4.796	4.322	1-hexadecene	0.697	−1.589	−3.144	−4.811	4.382
0.183	0.294	−0.134	−2.801	−4.291	4.180	1,2-dichloroethane	0.279	−0.091	−2.743	−4.314	4.274
0.222	0.273	−0.569	−2.918	−4.883	4.456	1-chlorobutane	0.255	−0.517	−2.848	−4.911	4.570
0.319	0.102	−0.187	−3.058	−4.090	4.324	dichloromethane	0.076	−0.112	−2.957	−4.130	4.488
0.191	0.105	−0.403	−3.112	−3.514	4.395	chloroform	0.089	−0.358	−3.051	−3.538	4.493
0.199	0.523	−1.159	−3.560	−4.594	4.618	carbon tetrachloride	0.507	−1.112	−3.497	−4.619	4.721
0.395	−0.094	−0.594	−1.280	−1.274	3.088	trifluoroethanol	−0.126	−0.501	−1.156	−1.323	3.291
0.350	0.358	−0.820	−0.588	−4.956	4.350	diethyl ether	0.330	−0.737	−0.478	−5.000	4.530
0.176	0.394	−0.985	−1.414	−5.357	4.524	dibutyl ether	0.380	−0.944	−1.358	−5.379	4.615
0.341	0.307	−0.817	−0.618	−5.097	4.425	methyl tert-butyl ether	0.280	−0.737	−0.510	−5.140	4.600
0.142	0.464	−0.588	−3.009	−4.625	4.491	benzene	0.452	−0.554	−2.964	−4.643	4.564
0.139	0.152	−0.374	−3.030	−4.601	4.540	fluorobenzene	0.140	−0.341	−2.985	−4.618	4.611
0.065	0.381	−0.521	−3.183	−4.700	4.614	chlorobenzene	0.375	−0.506	−3.161	−4.708	4.648
−0.017	0.436	−0.424	−3.174	−4.558	4.445	bromobenzene	0.437	−0.428	−3.178	−4.556	4.437
−0.192	0.298	−0.308	−3.213	−4.653	4.588	iodobenzene	0.313	−0.353	−3.272	−4.629	4.490
0.125	0.431	−0.644	−3.002	−4.748	4.524	toluene	0.421	−0.615	−2.962	−4.764	4.589
0.093	0.467	−0.723	−3.001	−4.844	4.514	ethylbenzene	0.459	−0.701	−2.971	−4.856	4.562
0.122	0.377	−0.603	−2.981	−4.961	4.535	m-xylene	0.367	−0.574	−2.941	−4.977	4.598
0.083	0.518	−0.813	−2.884	−4.821	4.559	o-xylene	0.511	−0.793	−2.857	−4.831	4.602
0.166	0.477	−0.812	−2.939	−4.874	4.532	p-xylene	0.463	−0.773	−2.886	−4.895	4.618
−0.196	0.537	0.042	−2.328	−4.608	4.314	nitrobenzene	0.552	−0.004	−2.388	−4.584	4.214
0.159	0.784	−1.678	−3.740	−4.929	4.577	cyclohexane	0.771	−1.640	−3.689	−4.949	4.659
0.023	−0.091	0.793	−1.463	−4.364	3.460	nitromethane	−0.093	0.799	−1.454	−4.368	3.472
0.246	0.782	−1.982	−3.517	−4.293	4.528	methylcyclohexane	0.762	−1.924	−3.439	−4.324	4.655
0.223	0.363	−0.384	−0.238	−4.932	4.450	THF	0.345	−0.332	−0.167	−4.960	4.565
0.123	0.347	−0.033	−0.582	−4.810	4.110	1,4-dioxane	0.337	−0.004	−0.542	−4.826	4.173
0.004	0.168	0.504	−1.283	−4.407	3.421	propylene carbonate	0.167	0.505	−1.281	−4.408	3.423
0.038	0.225	0.058	−0.976	−4.842	4.315	cyclohexanone	0.222	0.067	−0.963	−4.847	4.335
0.147	0.532	0.225	0.840	−4.794	3.674	N-methylpyrrolidinone	0.520	0.260	0.887	−4.813	3.750
0.056	0.332	0.257	1.556	−5.035	3.983	N-methyl-2-piperidone	0.327	0.271	1.575	−5.044	4.012
−0.032	0.696	−0.062	0.014	−4.092	3.405	N-formylmorpholine	0.698	−0.069	0.005	−4.089	3.389
0.097	0.285	0.059	−1.605	−4.562	4.028	benzonitrile	0.277	0.082	−1.574	−4.575	4.078
0.413	0.077	0.326	−1.566	−4.391	3.364	acetonitrile	0.044	0.423	−1.436	−4.443	3.576
−0.270	0.578	−0.511	0.715	−2.619	2.729	ethylene glycol	0.599	−0.575	0.631	−2.585	2.591
0.313	0.312	−0.121	−0.608	−4.753	3.942	acetone	0.287	−0.047	−0.509	−4.792	4.103
0.246	0.256	−0.080	−0.767	−4.855	4.148	butanone	0.236	−0.022	−0.689	−4.886	4.275
−0.194	0.327	0.791	1.260	−4.540	3.361	DMSO	0.342	0.746	1.200	−4.517	3.262
0.047	0.686	−0.943	−3.603	−5.818	4.921	carbon disulfide	0.682	−0.932	−3.587	−5.825	4.946
0.000	0.147	0.601	−0.381	−4.541	3.290	sulfolane	0.147	0.601	−0.380	−4.542	3.290
0.022	0.350	−0.432	0.708	−4.725	4.192	tributyl phosphate	0.544	−0.761	−0.966	−4.374	4.087
0.574	0.715	−1.027	−1.296	−4.512	3.446	peanut oil	0.670	−0.892	−1.121	−4.582	3.744

Original on the left, with *c* = 0 on the right.

One way to measure the effect of making *c* = 0 is to evaluate how the values of each solute-solvent term change as measured against the average solute descriptors (E_ave_ = 0.884, S_ave_ = 1.002, A_ave_ = 0.173, B_ave_ = 0.486, V_ave_ = 1.308). By multiplying the average absolute deviation of the solvent coefficients and the mean solute descriptor value, e.g. AAE(v) * Mean(V_ave_), the coefficients shifted from greatest to least in the following order v (0.124), s (0.043), e (0.013), b (0.011), a (0.010).

## Results and discussion

### Modeling

We calculated CDK descriptors for each solvent using the cdkdescui [30] and then created five random forest models for e_0_, s_0_, a_0_, b_0_, and v_0_ using R. The resulting models had out of bag (OOB) R^2^ values ranging between the barely significant 0.31 for e_0_ to the very seignificant 0.92 for a_0_, see the Open Notebook page for more details [29]. It is important to note that due to the limited number of data points, we decided not to split the data into training and test sets and instead use the OOB values which are automatically generated with random forest models as our means of validation. A summary of the modeling results can be found in Table 2.

**Table 2:** **Summary of statistical measures of the results of modeling**

**Model**	**N**	**OOB RMSE**	**OOB R** ^**2**^	**RMSE**	**R** ^**2**^	**Most significant descriptor**
e_0_	89	0.181	0.308	0.074	0.885	XLogP
s_0_	89	0.326	0.768	0.135	0.960	XLogP
a_0_	89	0.477	0.919	0.205	0.985	nHBAcc
b_0_	89	0.471	0.474	0.203	0.903	khs.sOH
v_0_	89	0.228	0.627	0.122	0.933	TopoPSA

Quite why some endpoints are more difficult to model than others is not known. Comparing the OOB R^2^ values with the standard deviation of the endpoints (e_0_: 0.31, s_0_: 0.77, a_0_: 0.92, b_0_:0.47, and v_0_: 0.63) we see no negative correlation between the range of a given endpoint and the actual prediction performances of the associated models as one would possibly suspect. It is our conjecture that as more measured values become available that refined models will have better performance. For now, these models should be used only as an initial starting point for exploring the wider solvent chemical space.

Errors in the predications of the coefficients for new solvents are not equivalent because when used to predict partition coefficients they are scaled by their corresponding Abraham descriptors, see equation 3. Thus, on average, when predicting solvent coefficients for new solvents, the errors in predicting v and s are more significant that errors in predicting a and b due to the difference in the sizes of average values for the solute descriptors. Multiplying the OOB-RMSE for each coefficient by the corresponding average descriptors value we see the following scaled RMSE values for e_0_, s_0_, a_0_, b_0_, and v_0_ of 0.16, 0.33, 0.08, 0.23, and 0.30 respectively. Thus the poor OOB R^2^ values for e_0_ (0.31) and b_0_ (0.47) seem not to be as detrimental to the applicability of the model as suggested by a first glance.

**Figure 1:**
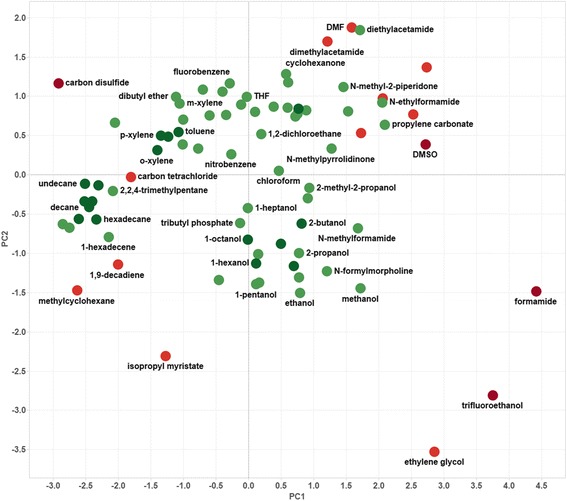
**Performance of the models on the existing chemical space of solvents with known coefficients.** The red color indicates poor performance – model outliers.

To analyze the modeling results further and to investigate model outliers we calculated an adjusted error D, the distance between the observed values and the predicted values scaled by the average descriptor values, for each solvent using the following equation:

4 $$ D=\sqrt{{\left({e}_0-{e}_0^p\right)}^2{A}_{ave}^2+{\left({s}_0-{s}_0^p\right)}^2{S}_{ave}^2+{\left({a}_0-{a}_0^p\right)}^2{A}_{ave}^2+{\left({b}_0-{b}_0^p\right)}^2{B}_{ave}^2+{\left({v}_0-{v}_0^p\right)}^2{V}_{ave}^2} $$

where the superscript p indicates the predicted value. These distances were then plotted as colors on a graph with the x and y axes corresponding to the first two principal components of the measured values for e_0_, s_0_, a_0_, b_0_, and v_0_, see Figure 1. Those solvents colored red have higher calculated distances between their measured and predicted values [Figure 1].

As we can see from the figure, model outliers include: formamide, trifluoroethanol, carbon disulfide, and DMSO. These solvents are on the outskirts of the chemical space. In fact, we can clearly see that the model makes far better predictions for solvents towards the center of the chemical space with particular success in predicting the coefficients for series such as alkanes and alcohols. These observations should give us caution when using the models to predict the solvent coefficients for novel solvents, especially when they do not lie within the chemical space established by solvents with known coefficients.

These Open Models (CC0) can be downloaded from the Open Notebook pages [29,31] and can be used to predict the solvent coefficients for any organic solvent; either with the view of predicting partition coefficients or other partitioning processes including solubilities via equation (1); or with the view of finding replacement and novel solvents for current syntheses, recrystallization procedures, and other solvent dependent processes [32]. As an informational note we remind readers that solute solubility and partitioning are only two of the considerations in finding an appropriate replacement solvent. Other considerations include the toxicity and the purchase price of the solvent, disposal costs of the solvent, physical properties of the solvent, and whether or not the solvent undergoes any undesired chemical reactions with other chemical compounds that might be present in the solution. For example, some chemical reactions take place at elevated temperatures and here one would want to use a solvent having a sufficiently high boiling point temperature that it would not vaporize under the experimental conditions.

### Sustainable solvents

As an example of the application of our models, we used our models to calculate the solvent descriptors for a list of sustainable solvents from a paper by Moity *et. al.* [33]. The resulting coefficients for 119 select novel sustainable solvents are presented in Table 3. A complete set of coefficients for all 293 solvents (sustainable, classic, and measured) can be found in Additional file 2. These values should be used in light of the limitation of the model as described above, as possible starting places for further investigation, and not as gospel.

**Table 3:** **Predicted solvent coefficients for select sustainable solvents**

**Solvent**	**e**_**0**_	**s**_**0**_	**a**_**0**_	**b**_**0**_	**v**_**0**_
1,3-dioxan-5-ol	0.407	−0.238	−0.110	−3.616	3.523
1,3-dioxolane	0.311	−0.233	−0.305	−4.661	4.029
1,3-dioxolane-4-methanol	0.404	−0.250	−0.108	−3.641	3.528
1,4-cineol	0.397	−0.616	−0.909	−4.718	4.299
1,8-cineol	0.393	−0.581	−0.921	−4.723	4.316
2-butoxy-1,3-propanediol	0.452	−0.493	−0.285	−3.531	3.826
2-furfuraldehyde	0.300	0.023	−0.539	−4.305	3.885
2-methyltetrahydrofuran	0.344	−0.557	−0.565	−4.686	4.440
2-pyrrolidone	0.306	0.011	0.734	−4.709	4.020
3-hydroxypropionic acid	0.324	−0.155	−0.180	−3.758	3.500
3-methoxy-3-methyl-1-butanol	0.310	−0.637	−0.200	−3.916	4.002
5-(hydroxymethyl)furfural	0.413	0.084	−0.314	−3.451	3.729
acetic acid	0.193	0.016	−0.103	−3.625	3.565
acetyl tributyl citrate	0.689	−0.837	−1.091	−4.415	3.969
alpha-pinene	0.544	−1.225	−3.200	−4.719	4.511
alpha-terpineol	0.410	−0.853	−0.527	−4.003	4.172
benzyl alcohol	0.365	−0.399	−0.381	−3.949	4.143
benzyl benzoate	0.483	−0.550	−1.155	−4.526	4.072
beta-farnesen	0.576	−1.418	−3.214	−4.852	4.470
beta-myrcene	0.572	−1.421	−3.173	−4.904	4.599
beta-pinene	0.543	−1.245	−3.217	−4.723	4.511
beta-terpineol	0.439	−0.807	−0.554	−4.038	4.158
butyl laurate	0.617	−0.934	−1.210	−4.578	4.108
butyl myristate	0.635	−0.922	−1.210	−4.541	4.108
butyl palmitate	0.623	−0.917	−1.210	−4.541	4.106
butyl stearate	0.615	−0.917	−1.208	−4.538	4.106
caprylic acid diethanolamide	0.476	−0.532	−0.279	−3.717	3.864
cyclademol	0.463	−1.076	−0.413	−4.012	4.182
cyclopentyl methyl ether	0.385	−0.387	−0.654	−4.717	4.470
decamethylcyclo-pentasiloxane	0.460	−0.728	−0.788	−4.318	3.963
dibutyl sebacate	0.680	−0.892	−1.239	−4.329	3.976
diethyl adipate	0.359	−0.384	−0.954	−4.515	4.025
diethyl glutarate	0.349	−0.308	−0.979	−4.477	4.021
diethyl phthalate	0.444	−0.397	−1.082	−4.412	4.016
diethyl succinate	0.354	−0.169	−0.897	−4.480	3.956
dihydromyrcenol	0.479	−1.150	−0.458	−4.025	4.272
diisoamylsuccinate	0.571	−0.700	−1.006	−4.286	3.973
diisobutyl adipate	0.464	−0.607	−0.997	−4.338	4.007
diisobutyl glutarate	0.481	−0.543	−1.019	−4.301	4.000
diisobutyl succinate	0.424	−0.430	−1.007	−4.326	3.982
diisooctyl succinate	0.711	−0.861	−1.127	−4.262	3.967
dimethyl 2-methylglutarate	0.344	−0.144	−0.783	−4.365	3.879
dimethyl adipate	0.345	−0.156	−0.875	−4.446	3.878
dimethyl glutarate	0.342	−0.099	−0.855	−4.422	3.849
dimethyl isosorbide	0.353	−0.132	−0.394	−4.083	3.587
dimethyl phthalate	0.411	−0.189	−1.005	−4.385	3.940
dimethyl succinate	0.337	0.063	−0.704	−4.430	3.830
dioctyl succinate	0.701	−0.891	−1.233	−4.298	3.966
dipropyleneglycol	0.392	−0.442	−0.225	−3.468	3.748
d-limonene	0.558	−1.298	−3.188	−4.832	4.527
ethyl lactate	0.242	−0.026	−0.412	−3.575	3.868
ethyl laurate	0.590	−0.902	−1.133	−4.660	4.124
ethyl linoleate	0.531	−0.837	−1.094	−4.519	4.129
ethyl linolenate	0.535	−0.838	−1.100	−4.522	4.135
ethyl myristate	0.601	−0.934	−1.168	−4.585	4.116
ethyl oleate	0.577	−0.885	−1.120	−4.520	4.114
ethyl palmitate	0.630	−0.922	−1.179	−4.544	4.111
ethylhexyllactate	0.515	−0.690	−0.821	−3.643	3.974
furfuryl alcohol	0.351	−0.195	−0.047	−3.553	3.880
gamma-valerolactone	0.293	0.151	−0.795	−4.521	3.957
geraniol	0.435	−0.953	−0.428	−4.113	4.238
geranyl acetate	0.517	−0.799	−1.124	−4.625	4.128
glycerol	0.405	−0.430	0.076	−3.421	3.476
glycerol carbonate	0.282	0.082	−0.587	−3.530	3.529
glycerol triacetate	0.325	−0.139	−0.913	−4.381	3.893
glycerol-1,2,3-tributyl ether	0.542	−0.934	−0.994	−4.257	4.082
glycerol-1,2,3-triethyl ether	0.370	−0.473	−0.778	−4.427	4.078
glycerol-1,2,3-trimethyl ether	0.315	−0.358	−0.407	−4.280	3.931
glycerol-1,2-dibutyl ether	0.437	−0.680	−0.624	−3.709	3.983
glycerol-1,2-diethyl ether	0.361	−0.415	−0.244	−3.663	3.932
glycerol-1,2-dimethyl ether	0.338	−0.410	−0.121	−3.525	3.663
glycerol-1,3-Dibutyl ether	0.423	−0.658	−0.583	−3.592	4.001
glycerol-1,3-diethyl ether	0.357	−0.398	−0.255	−3.555	3.864
glycerol-1,3-dimethyl ether	0.324	−0.402	−0.131	−3.467	3.676
glycerol-1-ethyl monoether	0.424	−0.380	−0.172	−3.432	3.583
glycerol-1-methyl monoether	0.394	−0.376	−0.106	−3.403	3.510
glycerol-2-ethyl monoether	0.435	−0.400	−0.151	−3.430	3.579
glycerol-2-methyl monoether	0.403	−0.429	−0.108	−3.382	3.500
glycofurol (n = 2)	0.479	−0.420	−0.427	−3.354	3.673
isoamyl acetate	0.310	−0.358	−0.830	−4.754	4.262
isobutyl acetate	0.251	−0.237	−0.798	−4.771	4.249
isododecane	0.631	−1.656	−3.473	−4.842	4.548
isopropyl palmitate	0.730	−0.984	−1.332	−4.354	4.040
isopropylacetate	0.232	−0.186	−0.803	−4.708	4.234
isosorbide dioctanoate	0.618	−0.827	−1.092	−4.216	3.888
menthanol	0.485	−1.103	−0.435	−4.031	4.184
menthanyl acetate	0.568	−0.685	−1.121	−4.472	4.094
menthyl acetate	0.566	−0.697	−1.117	−4.508	4.107
methyl 5-(dimethylamino) 2-methyl-oxopentanoate	0.323	−0.119	−0.405	−4.378	3.848
methyl abietate	0.635	−0.720	−1.152	−4.450	4.083
methyl laurate	0.535	−0.858	−1.071	−4.676	4.090
methyl linoleate	0.505	−0.806	−1.028	−4.524	4.081
methyl linolenate	0.510	−0.794	−1.023	−4.523	4.097
methyl myristate	0.583	−0.885	−1.110	−4.645	4.078
methyl oleate	0.572	−0.852	−1.072	−4.523	4.078
methyl palmitate	0.611	−0.890	−1.127	−4.578	4.076
methyl ricinoleate	0.578	−0.808	−0.890	−3.915	3.979
methyl stearate	0.591	−0.880	−1.121	−4.554	4.074
N,N-diethylolcapramide	0.485	−0.640	−0.330	−3.718	3.857
N,N-dimethyldecanamide	0.563	−0.767	−0.035	−4.812	4.103
N,N-dimethyloctanamide	0.484	−0.549	−0.010	−4.843	4.121
nopol	0.365	−0.784	−0.385	−4.026	4.112
n-propyl acetate	0.299	−0.349	−0.738	−4.889	4.267
oleic acid	0.485	−0.817	−0.611	−4.106	4.042
p-cymene	0.564	−1.163	−3.112	−4.797	4.526
PEG 200	0.490	−0.423	−0.310	−3.297	3.495
PEG 600	0.469	−0.528	−0.309	−3.307	3.502
perfluorooctane	0.386	−0.813	−2.663	−4.033	4.079
propionic acid	0.207	−0.105	−0.185	−3.981	3.840
1,2-propylene glycol	0.387	−0.447	0.259	−3.447	3.586
ricinoleic acid	0.477	−0.812	−0.787	−3.938	3.971
solketal	0.297	−0.208	−0.251	−3.678	3.789
terpineol acetate	0.470	−0.618	−1.089	−4.541	4.097
terpinolene	0.544	−1.209	−3.212	−4.860	4.535
tetrahydrofurfurylic alcohol	0.433	−0.365	−0.168	−3.544	3.857
tributyl citrate	0.572	−0.723	−0.887	−3.892	3.961
triethyl citrate	0.379	−0.317	−0.618	−3.835	3.826
trimethylene glycol	0.434	−0.627	0.236	−3.726	3.600

By comparing the predicted solvent coefficients to that of solvents with measured coefficients, we can make solvent replacement suggestions both in general and in particular. In general, the distance between solvents can be measured as the difference in predicted solubilities for the average compound.

5 $$ d= log\ {P}_1- log\ {P}_2= log\ {S}_1- log\ {S}_2 $$

6 $$ d=\left({e}_{01}-{e}_{02}\right)*{E}_{ave}+\left({s}_{01}-{s}_{02}\right)*{S}_{ave}+\left({a}_{01}-{a}_{02}\right)*{A}_{ave}+\left({b}_{01}-{b}_{02}\right)*{B}_{ave}+\left({v}_{01}-{v}_{02}\right)*{V}_{ave} $$

**Figure 2:**
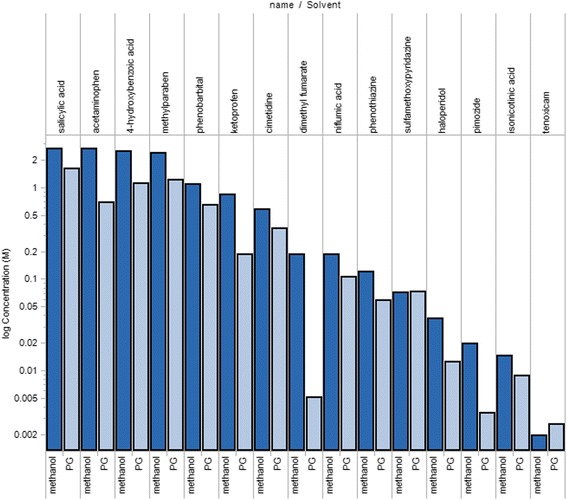
**Experimental solubilities in both methanol and 1,2-propylene glycol.**

Using this method we found several possible replacements. For example, 1,2-propylene glycol (e_0_ = 0.387, s_0_ = −0.447, a_0_ = 0.259, b_0_ = −3.447, v_0_ = 3.586) and methanol (e_0_ = 0.312, s_0_ = −0.649, a_0_ = 0.330, b_0_ = −3.355, v_0_ = 3.691) have a d-value of 0.07. This suggests that 1,2-propylene glycol may be a general sustainable solvent replacement for methanol. To confirm our model’s suggestion, we compared the solubilities of compounds from the Open Notebook Science Challenge solubility database [34] that had solubility values for both 1,2-propylene glycol and methanol, see [Figure 2].

Examining Figure 2, we see that solubility values are of the same order in most cases. The biggest discrepancy being for dimethyl fumerate. The measured solubility values are reported to be 0.182 M and 0.005 M for methanol and propylene glycol respectively [34], whereas the predicted solubilities are 0.174 M for methanol and 0.232 M for propylene glycol based upon the Abraham descriptors: E = 0.292, S = 1.511, A = 0.000, B = 0.456, V = 1.060 [35]. This suggests that the reported value for the solubility of dimethyl fumerate in ethylene glycol may be incorrect and that, in general, 1,2-propylene glycol is a sustainable solvent replacement for methanol.

Other strongly suggested general replacements include: dimethyl adipate for hexane, ethanol/water(50:50)vol for o-dichlorobenzene, and alpha-pinene for 1,1,1-trichloroethane. Many more replacement suggestions can be generated by this technique.

In a similar manner to the above procedure for general solvent replacement for all possible solutes, one can easily compare partition and solvation properties across all solvents for a specific solute (or set of solutes) with known or predicted Abraham descriptors (E, S, A, B, V). For example, using descriptors E = 0.730, S = 0.90, A = 0.59, B = 0.40, V = 0.9317 for benzoic acid (and using d = 0.001), we can make several benzoic acid-specific solvent replacement recommendations, see Table 4. These replacement suggestions do not seem unreasonable chemically and several examples can be explicitly verified by comparing actual measured solubility values [34]. Such a procedure can easily be done for other specific compounds with known or predicted Abraham descriptors to find alternative green solvents in varying specific circumstances (solubility, partition, etc.).

**Table 4:** **Replacement solvent suggestions for procedures involving benzoic acid**

**Solvents**	**Possible replacement**
benzonitrile	1,4-cineol
1-hexanol	N,N-diethylolcapramide
sulfolane	ethylene glycol
methyl tert-butyl ether	diethyl succinate
diethyl ether	ethylhexyllactate
2-pentanol	gamma-valerolactone
2-methyl-2-propanol	glycerol, cyclopentyl methyl ether
trifluoroethanol	methyl ricinoleate
diphenyl ether	isopropyl palmitate
n-propylamine	ricinoleic acid
propionitrile	1,4-cineol
ethylenediamine	diisobutyl adipate
2,6-dimethylpyridine	ethyl acetate
4-picoline	ethanol/water(80:20)vol
diethylamine	glycerol-1,2,3-triethyl ether, dihydromyrcenol
morpholine	ethanol/water(90:10)vol
diethylene glycol	glycerol-1-ethyl monoether
2-aminoethanol	glycerol-1-methyl monoether
3-pentanol	glycerol-2-methyl monoether
aniline	dimethyl phthalate
2-methyl-2-butanol	2-furfuraldehyde
nitroethane	butyl myristate

In addition to sustainable solvents, we also considered the list of commonly used solvents in the pharmaceutical industry [36]. Of all the solvents listed, the only one not covered previously by this work (Additional file 2) was 4-methylpent-3-en-2-one which has SMILES: O = C(\\C = C(/C)C)C and predicted solvent coefficients: e_0_ = 0.269, s_0_ = −0.362, a_0_ = −0.610, b_0_ = −4.830, v_0_ = 4.240.

## Conclusions

We have provided a set of Open Models that can be used to predict the Abraham coefficients for any organic solvent. These coefficients can then in turn be used to predict various partition processes and solubilities of compounds with known or predicted Abraham descriptors. We illustrated the usefulness of the models by demonstrating how one can compare solvent coefficients both in general and in particular for specific solutes or sets of solutes to find solvent replacement leads.

## Additional files

Additional file 1:
**Current list of dry solvents with known Abraham descriptors together with their c = 0 predicted values, SMILES, melting points, boiling points, and ChemSpider ID (CSID).**
Additional file 2:
**Predicted solvent coefficients for all 293 solvents considered in this study: sustainable, classic, and measured.**

## Abbreviations

LFER: Linear free energy relationship
CDK: Chemistry development kit
AAE: Average absolute error
OOB: Out of bag
DMF: Dimethyl formamide
THF: Tetrahydrofuran
DMSO: Dimethyl sulfoxide
PEG: Polyethylene glycol
SMILES: Simplified molecular-input line-entry system
CSID: ChemSpider ID
ONS: Open Notebook Science

## References

[1] Sprunger LM, Achi SS, Pointer R, Acree WE, Abraham MH (2010). Development of Abraham model correlations for solvation characteristics of secondary and branched alcohols. Fluid Phase Equilibr.

[2] Sprunger LM, Achi SS, Pointer R, Blake-Taylor BH, Acree WE, Abraham MH (2009). Development of Abraham model correlations for solvation characteristics of linear alcohols. Fluid Phase Equilibr.

[3] Sprunger LM, Proctor A, Acree WE, Abraham MH, Benjelloun-Dakhama N (2008). Correlation and prediction of partition coefficient between the gas phase and water, and the solvents dry methyl acetate, dry and wet ethyl acetate, and dry and wet butyl acetate. Fluid Phase Equilibr.

[4] Abraham MH, Acree WE, Leo AJ, Hoekman D (2009). The partition of compounds from water and from air into wet and dry ketones. New J Chem.

[5] Abraham MH, Acree WE, Cometto-Muniz JE (2009). Partition of compounds from water and from air into amides. New J Chem.

[6] Abraham MH, Acree WE, Leo AJ, Hoekman D (2009). Partition of compounds from water and from air into the wet and dry monohalobenzenes. New J Chem.

[7] Abraham MH, Acree WE (2008). Comparison of solubility of gases and vapours in wet and dry alcohols, especially octan-1-ol. J Phys Org Chem.

[8] Sprunger LM, Achi SS, Acree WE, Abraham MH, Leo AJ, Hoekman D (2009). Correlation and prediction of solute transfer to chloroalkanes from both water and the gas phase. Fluid Phase Equilibr.

[9] Sprunger LM, Gibbs J, Acree WE, Abraham MH (2008). Correlation and prediction of partition coefficients for solute transfer to 1,2-dichloroethane from both water and from the gas phase. Fluid Phase Equilibr.

[10] Abraham MH, Zissimos AM, Acree WE (2003). Partition of solutes into wet and dry ethers; an LFER analysis. New J Chem.

[11] Abraham MH, Zissimos AM, Acree WE (2001). Partition of solutes from the gas phase and from water to wet and dry di-n-butyl ether: a linear free energy relationship analysis. Phys Chem Chem Phys.

[12] Acree WE, Abraham MH (2006). The analysis of solvation in ionic liquids and organic solvents using the Abraham linear free energy relationship. J Chem Technol Biotechnol.

[13] Abraham MH, Acree WE (2006). Comparative analysis of solvation and selectivity in room temperature ionic liquids using the Abraham linear free energy relationship. Green Chem.

[14] Abraham MH, Ibrahim A, Acree WE (2007). Air to liver partition coefficients for volatile organic compounds and blood to liver partition coefficients for volatile organic compounds and drugs. Eur J Med Chem.

[15] Abraham MH, Ibrahim A, Acree WE (2008). Air to brain, blood to brain and plasma to brain distribution of volatile organic compounds: linear free energy analyses. Eur J Med Chem.

[16] Abraham MH, Ibrahim A, Zhao Y, Acree WE (2006). A database for partition of volatile organic compounds and drugs from blood/plasma/serum to brain, and an LFER analysis of the data. J Pharm Sci.

[17] Abraham MH, Ibrahim A (2006). Air to fat and blood to fat distribution of volatile organic compounds and drugs: Linear free energy analyses. Eur J Med Chem.

[18] Abraham MH, Ibrahim A, Acree WE (2006). Air to muscle and blood/plasma to muscle distribution of volatile organic compounds and drugs: Linear free energy analyses. Chem Res Toxicol.

[19] Sprunger LM, Gibbs J, Acree WE, Abraham MH (2009). Linear free energy relationship correlation of the distribution of solutes between water and cetyltrimethylammonium bromide (CTAB) micelles. QSAR Comb Sci.

[20] Wilson A, Tian A, Dabadge N, Acree WE, Varfolomeev MA, Rakipov IT (2013). Enthalpy of solvation correlations for organic solutes and gases in dichloromethane and 1,4-dioxane. Struct Chem.

[21] Stephens TW, Chou V, Quay AN, Shen C, Dabadge N, Tian A (2014). Thermochemical investigations of solute transfer into ionic liquid solvents: Updated Abraham model equation coefficients for solute activity coefficient and partition coefficient predictions. Phys Chem Liquids.

[22] Abraham MH, Smith RE, Luchtefeld R, Boorem AJ, Luo R, Acree WE (2010). Prediction of solubility of drugs and other compounds in organic solvents. J Pharm Sci.

[23] Bradley J-C, Acree Jr. WE, Lang ASID. An open logP model based upon Abraham Descriptors. ONS Challenge. Open Notebook. [http://onschallenge.wikispaces.com/ADlogP001]

[24] Van Noort PCM (2012). Solvation thermodynamics and the physical–chemical meaning of the constant in Abraham solvation equations. Chemosphere.

[25] Grubbs LM, Saifullaha M, De La Rosa NE, Yea S, Achia SS, Acree WE (2010). Mathematical correlations for describing solute transfer into functionalized alkane solvents containing hydroxyl, ether, ester or ketone solvents. Fluid Phase Equilib.

[26] Sprunger LM, Achi SS, Acree WE, Abraham MH (2010). Reply to comments of Endo and Goss concerning “development of correlations for describing solute transfer into acyclic alcohol solvents based on the Abraham model and fragment-specific equation coefficients. Fluid Phase Equilibr.

[27] The Chemistry Development Kit [http://sourceforge.net/projects/cdk]

[28] Bradley J-C, Acree Jr. WE, Lang ASID. Compounds with known Abraham descriptors. *figshare* 2014*.* [http://dx.doi.org/10.6084/m9.figshare.1176994]

[29] Bradley J-C, Lang ASID. Solvent coefficients for alternative ‘safe’ solvents. ONS Challenge. Open Notebook. [http://onschallenge.wikispaces.com/AbrahamSolventsModel003]

[30] Guha R. CDK Descriptor UI. [https://github.com/rajarshi/cdkdescui]

[31] Lang ASID. ONS Models in R. ONS Challenge. Open Notebook. [http://onschallenge.wikispaces.com/ONSModels-R]

[32] Bradley J-C, Acree Jr. WE, Lang ASID. Solvent coefficients for sustainable solvents. ONS Challenge. Open Notebook. [http://onschallenge.wikispaces.com/AbrahamSolventsModel004]

[33] Moity L, Durand M, Benazzouz A, Pierlot C, Molinier V, Aubry J-M (2012). Panorama of sustainable solvents using the COSMO-RS approach. Green Chem.

[34] Bradley J-C, Guha R, Hooker B, Koch SJ, Lang ASID, Neylon C, et al.: Open Notebook Science Solubility Challenge. Open Notebook [http://onschallenge.wikispaces.com/]

[35] Bradley J-C, Lang ASID. Real-time prediction of Abraham descriptors by Chemspider ID for measured solubilities in the Open Notebook Science Challenge solubility database. Webservice. [http://showme.physics.drexel.edu/onsc/models/solventselector.php?csids=553171&limreact=0&limprod=0&bp=0&washes=0]

[36] Grodowska K, Parczewski A (2010). Organic solvents in the pharmaceutical industry. Acta Pol Pharm.

